# Anti-Inflammatory and Cardioprotective Effects of Tadalafil in Diabetic Mice

**DOI:** 10.1371/journal.pone.0045243

**Published:** 2012-09-21

**Authors:** Amit Varma, Anindita Das, Nicholas N. Hoke, David E. Durrant, Fadi N. Salloum, Rakesh C. Kukreja

**Affiliations:** Division of Cardiology, Department of Internal Medicine, VCU Pauley Heart Center, Virginia Commonwealth University, Richmond, Virginia, United States of America; Brigham and Women’s Hospital, United States of America

## Abstract

**Background:**

Insulin resistance impairs nitric oxide (NO) bioavailability and obesity promotes a state of chronic inflammation and damages the vascular endothelium. Phosphodiesterase-5 inhibitors restore NO signaling and may reduce circulating inflammatory markers, and improve metabolic parameters through a number of mechanisms. We hypothesized that daily administration of the PDE-5 inhibitor, tadalafil (TAD) will attenuate inflammation, improve fasting plasma glucose and triglyceride levels, body weight, and reduce infarct size after ischemia/reperfusion injury in obese, diabetic mice.

**Methods:**

Twenty leptin receptor null (*db/db*) mice underwent treatment with TAD (1 mg/Kg) or 10% DMSO for 28 days. Body weight and fasting plasma glucose levels were determined weekly. Upon completion, hearts were isolated and subjected to 30 min global ischemia followed by 60 min reperfusion in a Langendorff model. Plasma samples were taken for cytokine analysis and fasting triglyceride levels. Infarct size was measured using computer morphometry of tetrazolium stained sections. Additionally, ventricular cardiomyocytes were isolated and subjected to 40 min of simulated ischemia and reoxygenation. Necrosis was determined using trypan blue exclusion and LDH release assay and apoptosis was assessed by TUNEL assay after 1 h or 18 h of reoxygenation, respectively.

**Results:**

Treatment with TAD caused a reduction in infarct size in the diabetic heart (23.2±1.5 vs. 47.8±3.7%, p<0.01, *n* = 6/group), reduced fasting glucose levels (292±31.8 vs. 511±19.3 mg/dL, p<0.001) and fasting triglycerides (43.3±21 vs. 129.7±29 mg/dL, p<0.05) as compared to DMSO, however body weight was not significantly reduced. Circulating tumor necrosis factor-α and interleukin-1β were reduced after treatment compared to control (257±16.51 vs. 402.3±17.26 and 150.8±12.55 vs. 264±31.85 pg/mL, respectively; P<0.001) Isolated cardiomyocytes from TAD-treated mice showed reduced apoptosis and necrosis.

**Conclusion:**

We have provided the first evidence that TAD therapy ameliorates circulating inflammatory cytokines and chemokines in a diabetic animal model while improving fasting glucose levels and reducing infarct size following ischemia-reperfusion injury in the heart.

## Introduction

The prevalence of diabetes in the United States in increasing at an astronomical rate and the American Diabetes Association currently estimates that 8.3% of the population has diabetes and nearly 79 million people have insulin resistance and are at risk for developing diabetes [1]. Insulin resistance and diabetes lead to a number of micro- and macro- vascular insults leading to retinopathy, nephropathy and painful neuropathy and eventually more adverse complications such as atherosclerosis, coronary artery disease, and cerebrovascular disease. The increased incidence of these complications has been attributed to higher levels of inflammatory cytokines, chronic hyperglycemia leading to formation of advanced glycation end products (AGEs) and elevated levels of oxidative stress leading to endothelial dysfunction. There is data to suggest that reduced levels of nitric oxide (NO) within the vascular endothelium contributes to impaired insulin utilization in patients with insulin resistance [2]. Vascular NO is critical for normal vasodilatation and endothelial function, and impairment of NO bioavailability and the NO-cyclic guanosine monophosphate (cGMP)-cGMP-dependent protein kinases (PKG) signaling cascade can lead to endothelial dysfunction [3]. A number of clinical studies have shown that hyperglycemia and increased AGEs are key factors in potentiating vascular inflammation and increasing levels of reactive oxygen species (ROS) and oxidative stress [4], [5]. This vascular milieu of elevated inflammation, impaired NO bioavailability, and oxidative stress plays an integral role in the progression of atherosclerosis and subsequently acute coronary syndromes culminating in significant morbidity and mortality of the diabetic patient [6].

Recent animal studies with NO synthase (NOS) inhibitors and endothelial NOS (eNOS) gene, suggest that the NO signaling pathway may regulate and promote glucose uptake in myocytes [7]–[10]. Moreover, eNOS knockout mice had decreased oxygen consumption, increased weight gain and were resistant to insulin [11]. In addition, several studies have indicated that insulin resistance itself may impair NO release and damage the endothelium through mechanisms that are reciprocally interconnected [4], [5]. For example, chronic hyperglycemia has been shown to increase circulating levels of inflammatory cytokines, chemokines, and expression of intracellular adhesion molecule-1, hence contributing further to this pathognomonic state [12].

The PDE-5 inhibitors, including sildenafil (Viagra™), vardenafil (Levitra™), tadalafil (Cialis™) and avanafil (Stendra) have been approved by the Food and Drug Administration for the treatment of erectile dysfunction (ED). Sildenafil and tadalafil (TAD) have also been approved for the management of pulmonary arterial hypertension [13]. A number of preclinical studies from our laboratory have shown that PDE-5 inhibitors have a powerful protective effect against myocardial ischemia/reperfusion (I/R) injury [14]–[18], doxorubicin and post-MI heart failure [19]–[23]. Mechanistically, we showed that sildenafil protects the heart against I/R injury through increased expression of inducible NOS (iNOS)/eNOS [24], [25], activation of cGMP-dependent protein kinase, PKG [25], [26], phosphorylation of glycogen synthase kinase-β (GSK-3β) [26], opening of mitochondrial K_ATP_ channels [14]–[16], and hydrogen sulfide generation [27]. Chronic administration of PDE-5 inhibitors has been associated with increased persistent vascular and endothelial function by increasing the level of endothelial cGMP generated by activation of eNOS [24], [27]. In streptozotocin-induced diabetic rats, long-term administration of the PDE-5 inhibitor, DA-8159, prevented ED and preserved endothelial function [28]. In a similar model, 14 days of treatment with sildenafil improved vasorelaxation through enhanced endogenous NO signaling [29]. In addition, clinical studies have revealed a potential protective role of these compounds on endothelial function in short- and long-term assessments [30]. In a large meta-analysis, it was reported that endothelial dysfunction is a significant independent risk factor for cardiac death, myocardial infarction (MI), stroke and the need for coronary revascularization [31]. Diabetic patients with ED were at increased risk for silent coronary artery disease and ED was a powerful predictor of cardiovascular (CV) morbidity and mortality [32]. A recent epidemiological study provided evidence of a strong correlation between the risk factors associated with metabolic syndrome (i.e. obesity, elevated fasting glucose levels, dyslipidemia, hypertension) and urinary cGMP excretion, suggesting that a reduction of NO bioactivity concurs with these CV risk factors [33]. Moreover, genetic variations of eNOS gene influenced energy expenditure, severity of glucose intolerance and risk of developing type II diabetes [34]. Chronic (alternate-day) administration of TAD in men with ED had improved endothelial function as indicated by marked changes in serum markers of endothelial function, increased insulin levels and a robust decrease in the inflammatory marker, high sensitivity C-reactive protein (hs-CRP) [35]. Furthermore, both acute and chronic administration of sildenafil improved endothelial function in patients with type-2 diabetes as observed by improved flow-mediated dilatation of the brachial artery [36], [37].

The diabetic myocardium is especially vulnerable to I/R injury and studies on obese diabetic animals models with hyperinsulinemia have confirmed this finding [38], [39]. Although the underlying mechanisms remain to be fully elucidated, a number of hypotheses have been proposed. Diabetics and models of hyperglycemia exhibit significantly higher levels of ROS production which impair mitochondrial energy metabolism and readily lead to cardiomyocyte apoptosis [40]. Likewise, the diabetic heart preferentially uses free fatty acids as an energy source and this requires nearly 12% more oxygen per adenosine triphosphate generated than when glucose is utilized [41]. Similarly, during ischemia, the diabetic myocardium does not have the ability to switch to glucose utilization for energy as does normal ischemic myocardium [38], [41]. The activity of the nicotinamide adenine dinucleotide diphosphate (NADPH)-dependent enzyme aldose reductase is elevated in diabetes and following I/R injury, its activity is increased further which serves to reduce myocardial glycolysis, glucose oxidation and increases the formation of AGEs [42], [43]. Furthermore, chronically elevated glucose levels have shown to lead to ventricular hypertrophy and process which decreases the overall vascular density within the myocardium. This phenomenon causes the diabetic to be extremely susceptible to endocardial ischemia and I/R injury [38], [44]. Recently, cytoprotective cytokines such as interleukin (IL)-33 that are downregulated in diabetic models, have also shown to have some role in increasing the vulnerability of the myocardium to ischemic insult. Rui *et al* recently showed that activation of protein kinase C-β in diabetes contributes to IL-33 attenuation and increased I/R susceptibility [44].

Therefore based on this background information, we propose that PDE-5 inhibitors would be ideal candidates to treat insulin resistance, and inflammation while protecting the diabetic heart against I/R injury and MI-induced heart failure. Among the currently available PDE-5 inhibitors, TAD has the advantage because it is long acting whereas the durations of action of sildenafil and vardenafil are generally 4 to 8 h [45]. Moreover, TAD is a highly selective inhibitor of PDE with >10,000-fold selectivity for PDE-5 over PDE-1 to PDE-4 and approximately 700-fold selectivity for PDE-5 over PDE-6 [45]. TAD is also the only PDE-5 inhibitor whose activity is unaffected by food and has a relatively short time to onset of action (16–17 min). Accordingly, we investigated whether chronic TAD treatment will attenuate inflammation and improve metabolic parameters such as body weight and fasting blood glucose and triglyceride levels while reducing infarct size through ameliorating cellular death pathways such as apoptosis and necrosis. We used the *db/db* diabetic mouse which has a point mutation of the leptin receptor that produces a chronic diabetic state and mimics aspects of human type 2 diabetes, including obesity, fasting hyperglycemia and insulin resistance [46], [47]. Insulin resistance is the earliest phenotypic change in these mice, evident by 10–12 days of age, with glucose intolerance to oral glucose challenge and reduced hypoglycemic response to insulin injection by 8–12 weeks of age [48].

## Materials and Methods

### Animals

Twenty leptin receptor null, homozygous *db/db* mice (strain B6.Cg-m +/+Lepr^db^/J) were purchased at a mean age of 12 weeks and treated for 4 weeks. All animals were purchased from The Jackson Laboratory (Bar Harbor, ME), and had the same genetic background [49]. The animal experiment protocols were approved by the Institutional Animal Care and Use Committee of Virginia Commonwealth University. All animal experiments were conducted under the guidance on humane use and care of laboratory animals for biomedical research published by the National Institutes of Health (No. 85-23, Revised 1996).

### Protocol for Animal Experiments

**Figure 1:**
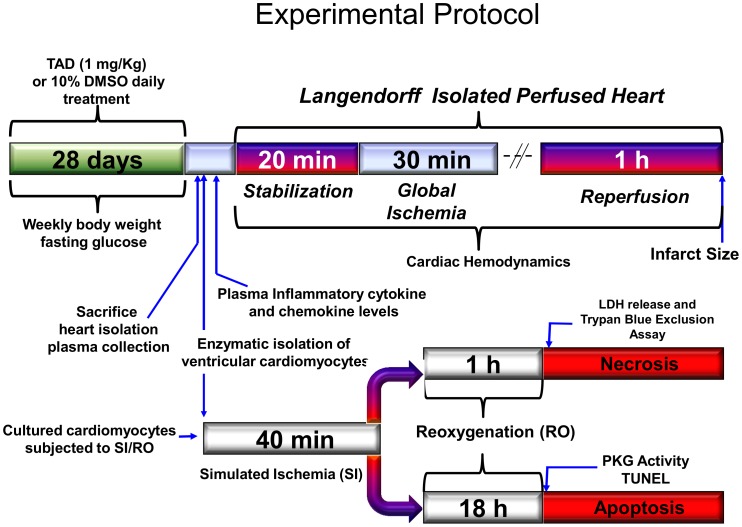
Experimental Protocol. Mice were treated with TAD (1 mg/Kg) or an equivalent volume of DMSO for 28 days. The hearts were, isolated and subjected to 20 min stabilization, followed by 30 min of no-flow global ischemiaand 60 min of reperfusion in a Langendorff model. At the time of sacrifice, serum samples were also collected for inflammatory cytokine and chemokine analysis. A second subset of *db/db* mice were treated and hearts were collected. The ventricular cardiomyocytes were isolated and subjected to 40 min of simulated ischemia (SI) followed by either 1 h or 18 h of reoxygenation (RO) for evaluation of necrosis and apoptosis as described under Methods.

Twelve *db/db* mice were randomized to receive daily intraperitoneal (*i.p.)* injections of TAD (1 mg/Kg in 10% dimethylsulfoxide [DMSO]) or daily *i.p.* injections of an equivalent volume of vehicle (10% DMSO in 0.9% NaCl) for 28 days as outlined in Fig. 1. The TAD dose was chosen based on interspecies dose extrapolation scaling to result in plasma concentrations equivalent to those found in humans receiving an oral dose of 20 mg/day. All mice had food (standard chow) and water provided *ad libitum*. Each animal had specific metabolic parameters monitored during the treatment period, including weekly fasting blood glucose levels and body weight. Upon completion of treatment, mice were anesthetized with pentobarbital sodium (70 mg/Kg, *i.p.*) and via thoracotomy, blood samples were taken by cardiac puncture for evaluating plasma inflammatory cytokines and chemokines.

### Langendorff Isolated Perfused Heart Preparation

The methods for the isolated, perfused mouse heart preparation have been previously described in detail [24]. In brief, mice treated with TAD or DMSO control group were anesthetized with pentobarbital sodium (100 mg/Kg) and heparin (33 units *i.p*.) and the hearts were quickly removed from the thorax and placed in a small dish containing ice-cold perfusate and heparin. The aortic opening was rapidly cannulated and tied on a 20-gauge blunt needle that was connected to a Langendorff perfusion system. After cannulation, the heart was retrogradely perfused at a constant pressure of 55 mm Hg with modified Krebs–Henseleit (K–H) solution containing (in mM) 118 NaCl, 24 NaHCO_3_, 2.5 CaCl_2_, 4.7 KCl, 1.2 KH_2_PO_4_, 1.2 MgSO_4_, 11 glucose, and 0.5 EDTA. The perfusion solution was continuously gassed with 95% O_2_+5% CO_2_ (pH ∼7.4) and warmed by a heating/cooling bath. The heart temperature was continuously monitored and maintained at 37°C throughout the experiment. Ventricular function was measured by a force-displacement transducer (model FT03, Grass) attached to the apex with a no. 5 surgical thread and a rigid metal hook. The resting tension of the isolated heart was adjusted to approximately 0.30 g. Ventricular developed force was continuously recorded with a PowerLab 8SP computerized data acquisition system connected to the force transducer. Coronary flow rate was calculated by timed collection of the perfusate. The hearts were not paced.

### Isolation of Adult Mouse Ventricular Cardiomyocytes

Four weeks after treatment with TAD or DMSO, 8 adult male leptin receptor null, homozygous *db/db* mice (strain B6.Cg-m +/+Lepr^db^/J) [49] were used to isolate ventricular cardiomyocytes. The ventricular cardiomyocytes were isolated using an enzymatic technique modified from the previously reported method [49]–[51]. In brief, the animal was anesthetized with pentobarbital sodium (100 mg/Kg, *i.p.*) and the heart was quickly removed. Within 3 min, the aortic opening was cannulated onto a Langendorff perfusion system and the heart was retrogradely perfused (37°C) at a constant pressure of 55 mmHg for approximately 5 min with a Ca^2+^-free bicarbonate-based buffer containing (in mM): 120 NaCl, 5.4 KCl, 1.2 MgSO_4_, 1.2 NaH_2_PO_4_, 5.6 glucose, 20 NaHCO_3_, 10 2, 3-butanedione monoxime, and 5 taurine, which was continuously bubbled with 95% O_2_+5% CO_2_. The enzymatic digestion was commenced by adding collagenase type II (Worthington, 0.5 mg/mL each) and protease type XIV (0.02 mg/mL) to the perfusion buffer and continued for ∼15 min. 50 µM Ca^2+^ was then added in to the enzyme solution for perfusing the heart for another 10–15 min. The digested ventricular tissue was cut into chunks and gently aspirated with a transfer pipette for facilitating the cell dissociation. The cell pellet was resuspended for a 3-step Ca^2+^ restoration procedure (i.e., 125, 250, 500 µM Ca^2+^). The freshly isolated cardiomyocytes were then suspended in minimal essential medium (Sigma catalogue# 6 M1018, pH 7.35–7.45) containing 1.2 mM Ca^2+^, 12 mM NaHCO_3_, 2.5% fetal bovine serum, and 1% penicillin–streptomycin. The cells were then plated onto the 35 mm cell culture dishes, which were pre-coated with 20 µg/ml mouse laminin in PBS +1% penicillin–streptomycin for 1 h. The cardiomyocytes were cultured in the presence of 5% CO_2_ for 1 h in a humidified incubator at 37°C, which allowed cardiomyocytes to attach to the dish surface prior to the experimental protocol.

### Glucose Measurements

Blood glucose concentrations were obtained using a handheld glucometer, (Lifescan, Milpitas, CA) using 5 µl of blood obtained from tail vein from 12 h fasted mice and applied directly to glucose test strips to measure fasting glucose levels on a weekly basis.

### Measurement of Plasma Inflammatory Cytokines and Chemokines

At the time of sacrifice, blood was collected by cardiac puncture into 2 separate ethylenediaminetetraacetic acid (EDTA) tubes, stored immediately on ice and centrifuged at 4°C, 3,000 *g* for 10 minutes. The serum and plasma were separated and stored at −80°C until analyzed. Plasma concentrations of representative cytokines: IL-1α, IL-1β, IL-2, IL-3, IL-5, IL-6, IL-8, IL-10, IL-12, IL-13, IL-17, TNF-α, interferon (IFN)-γ and chemokines: RANTES (Regulated upon Activation, Normal T-cell Expressed, and Secreted) also known as CCL (C-C chemokine ligand)-5, MIP-1α and -1β (macrophage inflammatory protein) which are also known as CCL-3 and CCL-4 was quantified using the Bio-Plex Pro magnetic cytokine assay (Bio-Rad, Hercules, CA). Plasma samples were assayed for triglycerides using commercially available colorimetric assay kits (Cayman Chemicals, Ann Arbor, MI).

### Infarct Size Assessment

At the end of reperfusion, the heart was immediately removed from the Langendorff apparatus, weighed and frozen at −20°C. The frozen heart was cut into six to seven transverse slices, stained by 10% tetrazolium chloride for 30 min at room temperature (∼22°C), and subsequently fixed with 10% formalin for 2 to 4 h. The infarct area was determined by computer morphometry by using Bioquant Imaging Software. The infarct size was presented as percentage of risk area.

### Cardiomyocytes Experimental Protocol

The cultured cardiomyocytes were subjected to simulated ischemia (SI) for 40 min by replacing the cell medium with an “ischemia buffer” which contained (in mM): 118 NaCl, 24 NaHCO_3_, 1.0 NaH_2_PO_4_, 2.5 CaCl_2_–2H_2_O, 1.2 MgCl_2_, 20 sodium lactate, 16 KCl, 10 2-deoxyglucose (pH adjusted to 6.2). In addition, the cells were incubated under hypoxic conditions at 37°C during the entire SI period by adjusting the tri-gas incubator to 1–2% O_2_ and 5% CO_2_. Reoxygenation (RO) was accomplished by replacing the ischemic buffer with normal medium under normoxic conditions. Assessment of cell necrosis was performed after 1 h after RO as seen in Fig. 1.

### Evaluation of Cell Viability and Apoptosis

Cell viability was assessed by trypan blue exclusion assay and by lactate dehydrogenase (LDH) release in the cell medium. At the end of protocol, 20 µL of 0.4% trypan blue (Sigma-Aldrich) was added into the culture dish. After approximately 5 min of equilibration, the cells were counted under microscope. For LDH measurements, the cellular medium was collected, and the enzyme activity was monitored spectrophotometrically using an assay kit (Sigma-Aldrich).

Cardiomyocyte apoptosis was evaluated by using terminal deoxynucleotidyl transferase-mediated dUTP nick-end labeling (TUNEL) with an ApopTag *in situ* apoptosis detection kit (Millipore Bioscience Research Reagents, Temecula, CA) according to the manufacturer’s instructions. The quantification of apoptosis was determined by counting the TUNEL-positive myocyte nuclei from five random fields per section and was expressed as percentage of total myocyte nuclei as previously reported [26].

### Protein Kinase G Activity

Protein kinase G activity was examined using a commercially available PKG activity kit (Cyclex; Nagano, Japan) in ventricular cardiomyocytes (*n* = 4/group). Activity was measured according to the manufacturer’s instructions. Spectrophotometric absorbance was measured at 450 nm. Results were normalized as per milligram of protein.

### Statistics

Continuous variables are expressed as mean ± standard error. Two-way analysis of variance (ANOVA) was used to compare pre- and post-intervention values between the 2 groups. Student’s *T* test was used for comparison of unpaired data between 2 groups and the one-way ANOVA to compare unpaired data between 3 or more groups. Discrete variables are expressed as percentage and the Chi-square or Fisher’s exact tests are used accordingly. Unadjusted two-tailed P values <0.05 are considered statistically significant.

## Results

### Effect of TAD on Fasting Plasma Glucose and Triglyceride Levels and Body Weight

**Figure 2:**
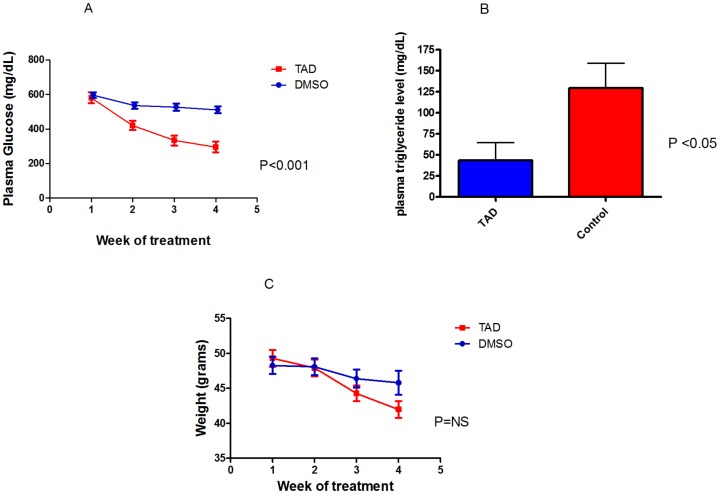
Effect of TAD on fasting blood glucose and body weight. The *db/db* mice were treated with TAD or DMSO as described in Figure 1 ***A:*** Fasting blood glucose levels. ***B***: Triglycerides. There was a significant decline in plasma triglyceride levels in the TAD group compared to control. ***C:*** Body weight. TAD treatment had significant effect in reducing blood glucose levels.

Fasting plasma glucose levels and body weight were evaluated weekly whereas plasma triglyceride levels were measured upon completion of therapy. The *db/db* mice treated with TAD showed a significant decrease in fasting plasma glucose levels (292±31.8 mg/dL vs. 511±19.3 mg/dL) compared to controls after the treatment period, [*n* = 6/group; Fig. 2A]. Similarly, there was a dramatic decline in fasting triglycerides in TAD treated *db/db* mice (43.3±21 vs. 129.7±29 mg/dL, p<0.05, *n* = 6; Fig. 2B). However, body weight remained unchanged between control *db/db* and TAD treated *db/db* mice (41.4±1.2 g vs. 45.8±1.7 g) [*n* = 6/group; Fig. 2C].

### Effect of TAD on Infarct Size

**Figure 3:**
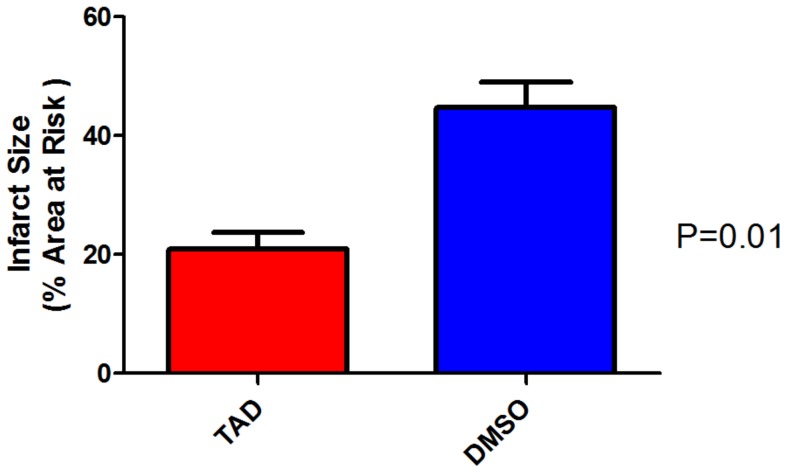
Effect of TAD on myocardial infarct size. Treatment with TAD significantly reduced infarct size when compared to the DMSO-treated control group, p = 0.011; *n* = 6/group.

**Figure 4:**
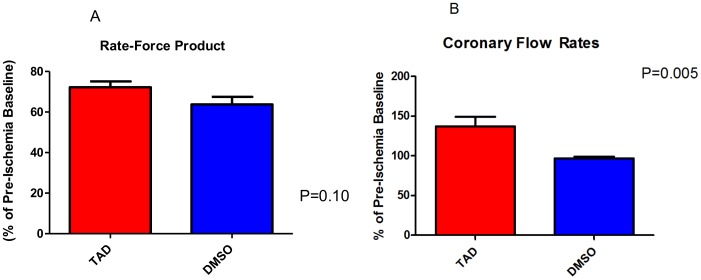
Cardiac function and coronary flow. ***A:*** Cardiac function presented as the double product of the heart rate and ventricular developed force (percentage of the pre-ischemic baseline) is shown. ***B:*** Coronary flow. The post-ischemic cardiac function remained unchanged whereas coronary flow was significantly improved in the TAD group (as a percentage of pre-ischemia baseline, p = 0.005).

Following I/R, infarct size was reduced after chronic treatment with TAD for 28 days as compared to vehicle (DMSO)-treated *db/db* mice (23.2±1.5% vs. 47.8±3.7%, p<0.01, *n* = 6/group; Fig. 3). There was no significant difference in the pre-ischemia basal functional parameters (i.e. developed force, rate-force product, and resting tension) between the two treatment groups (*n* = 6/group). The post-ischemic rate-force product (expressed as % of pre-ischemic baseline compared to the control group) was not changed (72.2±2.9% vs. 63.8±3.8%, p = 0.10, *n* = 6/group; Fig. 4A). However, the coronary flow rate improved in the TAD treated *db/db* mice (147.4±3.2% of the pre-ischemic baseline vs. 98.3±1.2% in the DMSO treated mice, p = 0.005, *n* = 6/group; Fig. 4B.) Baseline and post-ischemic coronary flow rates ranged from 1.8 to 2.1 mL/min and 1.7 to 1.9 mL/min in the TAD group, respectively and 1.7 to 2.0 mL/min and 1.5 to 1.6 mL/min in the control group, respectively.

### Effect of TAD on Cardiomyocyte Necrosis and Apoptosis

**Figure 5:**
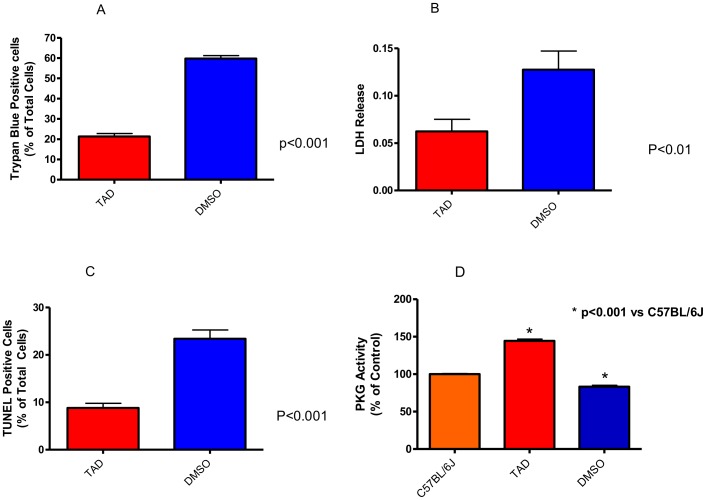
Effect of TAD in protection of ventricular cardiomyocytes against necrosis and apoptosis following simulated ischemia/reoxygenation. Necrosis was determined by trypan-positive (A) and LDH release in the medium (B); apoptosis was determined by TUNEL staining (C). TAD treated group had significantly lower trypan-blue positive cardiomyocytes and LDH release in the medium as compared with control (*n* = 4/group). Similarly, TAD treatment significantly reduced apoptotic cells when compared to DMSO, p<0.001, *n* = 4/group. (D): PKG activity. PKG activity was significantly higher in the TAD group, p<0.001, *n* = 4/group.

We further evaluated the effect of TAD treatment in protection of primary cardiomyocytes isolated from *db/db* mice against SI/RO injury. Our method of cell preparations yielded at least 85–90% of the cardiomyocytes with rod shape morphology. After 40 min of SI and 1 h of RO, the percent of trypan blue-positive (necrotic) cardiomyocytes was 59.8±1.5 in the control group. Treatment with TAD resulted in decrease of ∼64% of trypan blue-positive cardiomyocytes (i.e., 21.3±1.5%; p<0.001 vs. control, *n* = 4; Fig. 5A). Similarly, TAD treated cardiomyocytes demonstrated attenuated LDH release in the cell medium compared to control after SI/RO (p<0.01, *n* = 4; Fig. 5B). After 18h of RO, apoptosis was also inhibited as indicated by reduced number of TUNEL-positive cells in TAD treated cardiomyocytes (23.4±1.9% vs. 8.8±1% in the TAD treated group, (p<0.001, *n* = 4; Fig. 5C).

### Effect of TAD on Protein Kinase G Activity

TAD treated cardiomyocytes had a 44.3±2.5% increase in PKG activity compared to C57BL/6 non-treated controls whereas the DMSO treated group had a 16.7±1.8% decrease compared to the non-diabetic control, p<0.001, *n* = 4, Fig. 5D.

### Plasma Inflammatory Cytokine and Chemokine Levels

**Figure 6:**
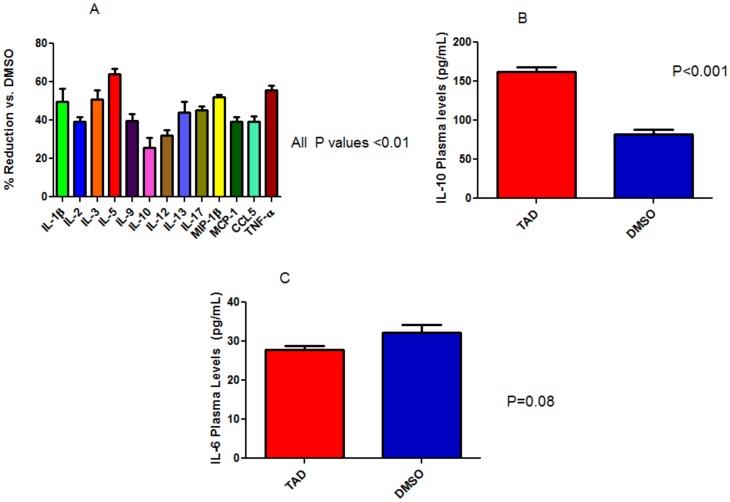
Effect of TAD on plasma inflammatory cytokines and chemokines. TAD treatment caused reductions in the cytokines IL-1β, TNF-α, IFN-γ, and chemokines MIP-1β, MCP-1, and RANTES (p<0.01, compared to DMSO; *n* = 6/group) as shown in ‘A”. The levels of IL-10 was higher in TAD treated mice than control, (p<0.001; *n* = 6/group) as shown in ‘B’. No significant changes in the IL-6 levels were observed between the two groups as shown in ‘C’.

Nearly all pro-inflammatory cytokines were decreased when measured after 28 days of treatment with TAD in the *db/db* mice when compared to the DMSO-treated controls [Fig. 6A]. Specifically, the anti-inflammatory cytokine IL-10 was increased in the TAD treated mice as compared to DMSO, (p<0.001, *n* = 6/group; Fig. 6B). TNF-α and IL-1β were significantly reduced compared to control (257±16.51 vs. 402.3±17.26 and 150.8±12.55 vs. 264±31.85 pg/mL, respectively; P<0.001), while IL-6 was not changed i.e, 27.8±1.1 pg/mL vs. 32.3±1.3 pg/mL, p = 0.08, *n* = 6/group; Fig. 6C. The chemokines RANTES (CCL-5), MIP-1β (CCL-4), and MCP-1 (CCL-2) were also reduced significantly [*n* = 6/group; Fig. 6A]. Chemokines MIP-1α (CCL-3) and Eotaxin, however, were not reduced significantly with TAD treatment.

## Discussion

In the present study, we showed for the first time that circulating levels of two key pro-inflammatory cytokines, TNF-α and IL-1β were significantly reduced after chronic TAD treatment. There was a trend towards a reduction in IL-6, whereas the anti-inflammatory cytokine IL-10 was significantly higher in TAD treated *db/db* mice compared to control. With an overall decreased inflammatory profile, improved fasting plasma glucose and triglyceride levels and a trend towards decreased body weight, our results correlate with previously published data and known physiological pathways, that IL-10 levels are inversely proportional to adiposity [52]. The adipose tissue of lean mice secretes an unusually high level of IL-10 which is shown to suppress inflammation. Furthermore, the number of T cells that secrete IL-10 dramatically decrease in proportion to increasing obesity [52].

Macrophage inflammatory protein-1β (MIP-1β), monocyte chemotactic protein-1 (MCP-1) and Regulation upon Activation, Normal T-cell Expressed and Secreted (RANTES) are three chemokines with the C-C ligand motif and play a significant role in the activation and proliferation of the inflammatory cascade, especially in adipose tissue and the endothelium [53]. Not surprisingly, TNF-α can activate MCP-1 and MIP-1β which further recruits macrophages to the adipose tissue hence potentiating the inflammation [54]. This invariably leads to the chronic low-level state of inflammation found in obesity. We found that along with reduced levels of TNF-α, the chemokines MIP-1β, MCP-1 and RANTES were all significantly reduced.

The role of IL-6 in insulin resistance has been controversial and sometimes paradoxical. Concentrations of IL-6 are increased in obesity and have been shown to predict the incidence of type 2 diabetes in individuals [55]. However, as hepatic and adipose production of IL-6 promote insulin resistance, IL-6 produced by skeletal muscle especially during intense exercise is beneficial [56]. Further adding to this controversy, mice with the genetic deletion of IL-6 have significant hepatic inflammation and develop insulin resistance [57]. Despite a slight reduction in circulating levels of IL-6 in the treatment group, the difference was not statistically significant.

Our results also show that TAD, similar to other PDE-5 inhibitors in non-diabetic models, significantly reduces infarct size following I/R in the diabetic heart and attenuates necrosis and apoptosis following SI/RO in isolated ventricular cardiomyocytes (Fig. 5A–C). Cardiac function did not improve significantly in the treatment group during isolated I/R, albeit there was a trend towards improved cardiac performance in addition to improved coronary flow rates. Using proteomic analysis, our laboratory recently demonstrated that chronic TAD treatment (28 day treatment) modulates cardiac proteins, specifically those associated with cytoskeletal rearrangement such as myosin light chain-2, myosin light chain-4, myosin heavy chain-α, and myosin binding protein-C [58]. These data suggest that TAD therapy may downregulate cytoskeletal contractile proteins associated with cardiac remodeling and heart failure. We also found that *in vivo* cardiac dysfunction was only seen after 24 weeks of age compared to the non-diabetic controls. This may explain why no significant improvement in cardiac function was noted after only 28 days of TAD treatment. In these experiments, the average age of *db/db* mice was approximately 16 weeks, which was too early to observe significant upregulation in cytoskeletal contractile proteins and consequent functional impairment of myocardial contraction.

It has been reported that leptin itself is protective after I/R injury through the induction of signal transducer and activator of transcription-3, which leads to downstream activation of cardioprotective genes [59], [60]. The leptin deficient mouse has impaired cardiac function after I/R injury in addition to higher cardiomyocyte death by apoptosis, ventricular hypertrophy and heart failure [60]. Apparently the lack of synthesis of cardioprotective genes in leptin knock-out mice may cause larger infarct size as compared to wild-type mice.

In the present study, although body weight was not significantly reduced, fasting plasma glucose levels was observed after 3 weeks of treatment. PKG activity was significantly increased in the TAD treated cardiomyocytes as compared to DMSO controls (Figure 5D). We propose that restoration of NO-sGC-PKG signalling following chronic treatment with TAD could affect insulin resistance and improve fasting blood glucose levels. Given the improved NO bioavailability and vasodilatation with PDE-5 inhibitors [61], there is increased blood flow for muscle glucose utilization and additionally, the decreased TNF-α in the circulation may potentially attenuate the amount of IRS-1 receptor phosphorylation and improve insulin signalling. It has also been postulated that chronic PDE-5 inhibitor treatment may increase fatty acid oxidation and may also be a potential mechanism for preventing insulin resistance. In a mouse model of diet-induced obesity and insulin resistance, chronic sildenafil treatment was able to improve insulin action and decrease body mass [2]. Moreover, it has been shown that prolonged treatment with TAD in C2C12 myoblasts improved oxidative capacity as demonstrated by increase in fatty acid metabolism, including the activities of citrate synthase and 3-OH acylCoA dehydrogenase [62]. One possible explanation is that PKG has been shown to affect insulin signaling and mitochondrial biogenesis in brown adipose tissue by inhibiting the activity of RhoA and Rho-associated kinase (ROCK), thereby relieving the inhibitory effects of ROCK on IRS-1 [63]. This allows the activation of the phosphotidyl-inositol-3-kinase (PI3K)-Akt signaling cascade downstream of the insulin receptor. In addition, Haas *et al* showed that PKG mediated the ability of NO and cGMP to induce mitochondrial biogenesis and increase the expression of uncoupling protein-1 (UCP-1), a protein necessary in energy expenditure through thermogenesis [63]. Similarly, NO and eNOS have been shown to directly correlate with mitochondrial biogenesis as the abundance of peroxisome proliferator activated receptor-gamma (PPAR-γ) [64], [65].

Oxidative stress mediated by hyperglycemia-induced generation of ROS contributes significantly to the development and progression of diabetes and related vascular damage [66]. Diabetes is usually accompanied by increased production of free radicals and impaired antioxidant mechanisms. Moreover, ROS are involved in insulin resistance via its regulatory effects on mitochondrial function [67], [68]. The diabetic vessels from animal models and endothelial derived cells in cell culture models under hyperglycemic conditions exhibit high levels of ROS associated with decreased levels of endothelial NO [69], [70]. The chronic treatment with TAD in diabetic mice was shown to improve redox signaling by enhancing the antioxidant enzyme glutathione *S*-transferase Kappa-1 (GSKT-1) and downregulate redox regulatory chaperones, heat shock protein 8 and 75 kDa glucose regulatory protein [58]. Moreover, TAD treated diabetic mice had significantly lower levels of GSSG/GSH suggesting reduction of oxidative stress [58].

In summary, we have provided evidence that TAD therapy ameliorates circulating inflammatory cytokines and chemokines in a diabetic model while improving fasting glucose levels and reducing infarct size following I/R injury in the heart. These results suggest that daily use of PDE-5 inhibitors may be a promising therapy for cardioprotection as well as reduction of pro-inflammatory cytokines in diabetic patients.
